# Cytotoxic capability and the associated proteomic profile of cell-free coelomic fluid extracts from the edible sea cucumber *Holothuria tubulosa* on HepG2 liver cancer cells

**DOI:** 10.17179/excli2022-4825

**Published:** 2022-04-25

**Authors:** Claudio Luparello, Rossella Branni, Giulia Abruscato, Valentina Lazzara, Laszlo Drahos, Vincenzo Arizza, Manuela Mauro, Vita Di Stefano, Mirella Vazzana

**Affiliations:** 1 Dipartimento di Scienze e Tecnologie Biologiche, Chimiche e Farmaceutiche (STEBICEF), Università di Palermo, Palermo, Italy; 2 MS Proteomics Research Group, Research Centre for Natural Sciences, Eötvös Loránd Research Network, Budapest, Hungary

**Keywords:** hepatocellular carcinoma, invertebrate, echinoderm, cell behavior, protein profile

## Abstract

Hepatocellular carcinoma (HCC) is an aggressive cancer histotype and one of the most common types of cancer worldwide. The identification of compounds that might intervene to restrain neoplastic cell growth appears imperative due to its elevated overall mortality. The marine environment represents a reservoir rich in bioactive compounds in terms of primary and secondary metabolites produced by aquatic animals, mainly invertebrates. In the present study, we determined whether the water-soluble cell-free extract of the coelomic fluid (CFE) of the edible sea cucumber *Holothuria tubulosa* could play an anti-HCC role *in vitro *by analyzing the viability and locomotory behavior, cell cycle distribution, apoptosis and autophagy modulation, mitochondrial function and cell redox state of HepG2 HCC cells. We showed that CFE causes an early block in the cell cycle at the G_2_/M phase, which is coupled to oxidative stress promotion, autophagosome depletion and mitochondrial dysfunction ultimately leading to apoptotic death. We also performed a proteomic analysis of CFE identifying a number of proteins that are seemingly responsible for anti-cancer effects. In conclusion, *H. tubulosa*'s CFE merits further investigation to develop novel promising anti-HCC prevention and/or treatment agents and also beneficial supplements for formulation of functional foods and food packaging material.

## Introduction

Hepatocellular carcinoma (HCC) is an aggressive cancer histotype accounting for about 90 % of primary liver tumor cases and is estimated as the sixth most common type of cancer worldwide and the fourth leading cause of cancer-related lethality in the recent years (Suresh et al., 2020[68]; Llovet et al., 2021[33]). Although there are multiple factors involved in the development of HCC, it usually follows the onset of hepatic cirrhosis from any etiology. Within this context, damaged hepatocytes are addressed to a chronic reparation program based on a high turnover level which ultimately results in the accumulation of genetic alterations and in progressive cell de-differentiation and modification of the hepatic microenvironment, supporting cell oncogenic transformation (Castelli et al., 2017[7]). HCC prognosis, which often gives rise to extensive metastasis and displays an elevated recurrence rate after resection or ablation, is generally poor and the overall mortality to incidence ratio is estimated to be close to one (McGlynn et al., 2021[49]). The advancement in knowledge of deranged intracellular signalling which leads to HCC development, has prompted the identification of compounds that might intervene on key aspects of the impaired pathways to inhibit neoplastic cell growth (Kumari et al., 2018[28] and references therein). However, an accurate biological characterization of the molecular mechanism of action of the agents under study is necessary for the development of targeted therapies.

The marine environment, which covers three-quarters of the globe, is rich in extraordinary biodiversity representing a resource of bioindicator organisms and a reservoir of bioactive compounds in terms of primary and secondary metabolites produced by aquatic animals (Chiaramonte et al., 2020[10]; Inguglia et al., 2020[23]; Mauro et al., 2020[47]; Vazzana et al., 2020[71][70]). Such molecules regulate very diverse biological activities to implement the necessary adaptation mechanisms for survival in strongly heterogeneous marine ecosystems that are comprised of complex habitats frequently exposed to extreme conditions. These marine-derived natural products have been shown to exert a wide range of therapeutic effects in humans, such as anti-microbial, anti-oxidant, anti-cancer, anti-inflammatory, wound healing and immunomodulatory effects, thereby interfering with the onset and progress of numerous pathogenic diseases (Senthilkumar and Kim, 2013[60]; Lazzara et al., 2019[29]; Luparello et al., 2020[41][40]; Luparello, 2021[38]). Based on the health benefits associated with marine bioactives, today's research is also prominently directed to investigate their potential utilization in food and supplement industries (Boziaris, 2014[6]; Suleria et al., 2015[65]; Mauro et al., 2020[46]; Punginelli, 2021[57]).

Among the marine invertebrate species, Holothurians (i.e. sea cucumbers) have been the object of several studies owing to their curative, nutraceutical and food values which are well-known from traditional folk medicine in several Asian countries (e.g. Correia-da-Silva et al., 2017[12]; Mauro et al., 2021[48]). *Holothuria tubulosa*, an edible sea cucumber species that is widely distributed in the Mediterranean sea including the coasts around Sicily, has been an object of investigations owing to the powerful anti-inflammatory activity of its methanolic extracts, due, at least in part, to the presence of fucoidan, and the anti-microbial activity of peptides contained in its immune cells, the coelomocytes (Herencia et al., 1998[20]; Schillaci et al., 2013[59]; Zhu et al., 2021[80]). Some research has also previously reported the anti-breast cancer effect of the cell-free aqueous extract of the coelomic fluid (CFE) obtained from this echinoderm (Luparello et al., 2019[43]). In the present study, we determined whether the CFE of *H. tubulosa* could produce beneficial results against tumors of the digestive apparatus, in particular playing an anti-hepatocarcinoma role, thus representing a novel potential anti-cancer agent deserving further in-depth investigation for functional food purposes. The cell line HepG2, derived from liver biopsies of a 15-year-old Caucasian male with differentiated hepatocellular carcinoma (Donato et al., 2015[15]), was chosen as the model system for this *in vitro *investigation and the effect of the CFE on the viability and locomotory behavior, cell cycle distribution, apoptosis and autophagy modulation, mitochondrial function and cell redox state was tested. Here we report that CFE-treated HepG2 cells are characterized by the inhibition of cell viability, the impairing of cell cycle progress, motile behavior, mitochondrial metabolism, reactive oxygen species (ROS) production, and autophagic flux, and the onset of apoptotic death. In addition, the data obtained from the proteomic analysis of the CFE identified a number of proteins potentially responsible for the observed impairment of biological activity in HepG2 cells. 

## Materials and Methods

### Catching and maintaining of the animals

**Figure 1:**
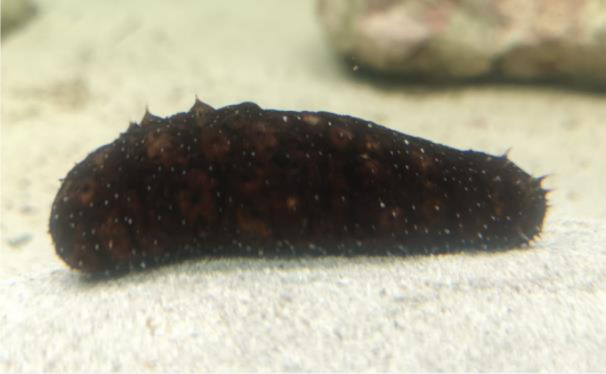
A *Holothuria tubulosa *sea cucumber specimen

A sample of 60 healthy adults of *H. tubulosa* sea cucumbers (Figure 1(Fig. 1)), displaying a length of 11 ± 0.98 cm and a body weight of 46 ± 7.5 g, was fished in the Gulf of Palermo (Sicily, Italy) at a depth of 5-10 m near a grassland of *Posidonia oceanica*. The animals were kept for acclimation in a constantly-aerated aquarium filled with artificial sea water (0.425 M NaCl; 9 mM KCl; 9.3 mM CaCl_2_·2H_2_O; 0.0255 M MgSO_4_·7H_2_O; 0.023 M MgCl_2_·6H_2_O; 2 mM NaHCO_3_; pH 8.0) at 15 ± 2 °C and fed with commercial invertebrate food (Algamac 3000, Aquafauna Bio-Marine Inc., Hawthorne, CA, USA). 

### Bleeding procedure and preparation of the CFE

The CFE was prepared as described by Luparello et al. (2019[43]). Briefly, the CF was collected from the animals after making an incision measuring 3-5 cm on the anterior-dorsal side using a scalpel. Samples were held at 4 °C before centrifugation at 1000 g for 10 min at 4 °C to remove coelomocytes. The cell-free CFE was subsequently lyophilized in an Alpha 2-4 LD plus freeze-dryer (Martin Christ, Osterode am Harz, Germany). Aliquots of the samples were resuspended in a minimum volume of sterile distilled water. The CFE protein concentration was evaluated in a Qubit 3.0 fluorometer using the Qubit Protein Assay Kit (ThermoFisher, Waltham, MA, USA) according to the manufacturer's instructions.

### Proteomic analysis

The CFE samples were dissolved in 0.25 % RapiGest (Waters Co., Milford, MA, USA) and passed through 10 kDa filters (keeping the retentate) to remove the RIPA buffer, and then the nominal protein concentration was measured for each sample using a NanoDrop 2000 UV-VIS spectrophotometer (ThermoFisher). Ten µg of each sample were reduced using RapiGest and dithiothreitol (ThermoFisher), and subsequently alkylated using iodoacetic acid (ThermoFisher) in 25 mM ammonium bicarbonate buffer (ThermoFisher). Samples were digested in-solution using LysC-trypsin (Mass Spec grade, Promega, Madison, WI, USA) at a 1:100 ratio, then trypsin at a 1:25 ratio. Proteolysis was stopped by adding formic acid (ThermoFisher), and the samples were dried down and cleaned-up using C18 spin columns (Thermo Scientific Sunnyvale, CA, USA) according to the manufacturer's protocol. The cleaned peptide extracts were dried down and stored at -20 °C until analysis.

Aliquots of 1 µg of the tryptic digests were analysed using a Dionex Ultimate 3000 nanoRSLC (Dionex, Sunnyvale, CA, USA) coupled to a Bruker Maxis II ETD mass spectrometer (Bruker Daltonics GmbH, Bremen, Germany) via CaptiveSpray nanobooster ion source. The samples were desalted by 0.1 % trifluoroacetic acid at a flow rate of 5 µL/min for 8 minutes using an Acclaim PepMap100 C-18 trap column (100 µm × 20 mm, Thermo Scientific). The peptides eluting from the precolumn were separated on an ACQUITY UPLC M-Class Peptide BEH C18 column (130 Å, 1.7 µm, 75 µm × 250 mm, Waters) at a 300 nL/min flow rate and 48 °C column temperature using a linear gradient from 4 % B to 50 % B in 120 minutes. Solvent A was 0.1 % formic acid, and solvent B was acetonitrile with 0.1 % formic acid. The cycle time for data-dependent acquisition, was 2.5 s. MS spectra were acquired at 3 Hz, while MS/MS spectra were acquired at 4 or 16 Hz, depending on the intensity of the precursor ion. Singly charged ions were excluded from the analysis.

The proteins were first identified by searching against the Uniprot Aechinodermata (downloaded: 07/03/2020) database using the Byonic software search engine (v3.8.13, Protein Metrics Inc, San Carlos, CA, USA) with the following parameters: 1 % FDR, 20 ppm peptide mass tolerance, 30 ppm fragment mass tolerance, 2 missed cleavages, trypsin as enzyme, carbamidomethylation of cysteines as fixed modification and following variable modifications (Oxidation/ +15.994915 @ M, Deamidated/+0.984016 @ N, Deamidated/+0.984016 @ Q, Gln->pyro-Glu/-17.026549 @ NTerm Q, Glu->pyro-Glu/ -18.010565 @ NTerm E). Protein hits were filtered by Scaffold (version 4.11, Proteome Software, Inc., USA) using the same parameters stated above in addition to the following parameters: Protein Grouping Strategy: Experiment-wide grouping with protein cluster analysis, Peptide Thresholds: 95.0 % minimum, Protein Thresholds: 1 % FDR and 2 peptides minimum. Subsequently, protein identification was also performed by BlastP comparison to non-redundant protein sequence and model organism databases (available at https://blast.ncbi.nlm.nih.gov/Blast.cgi?PAGE=Proteins; accessed in May 2021). An expected value < 1 was set as cutoff.

### Cell culture

The HepG2 HCC cell line was routinely grown in a high glucose-DMEM medium supplemented with 10 % fetal calf serum (Life Technologies, Carlsbad, CA/USA) and antibiotics (100 U/mL penicillin and 100 µg/mL streptomycin; Life Technologies), at 37 °C in a 5 % CO_2_ atmosphere.

### MTT assay

As an indicator of cytotoxicity, an MTT assay was used to measure cellular activity as reported by Longo et al. (2015[34]). Briefly, exponentially-growing HepG2 cells were seeded in 96-well plates at a concentration of 5500 cells/well and, after overnight adhesion, treatments with different concentrations of CF extract were applied for either 24 or 48 h. After the addition of MTT and cell solubilization, the absorbance of the dissolved formazan was measured in an automated microplate reader at a wavelength of 550 nm. The half maximal inhibitory concentrations (IC_50_) were evaluated with the Quest Graph IC_50_ calculator (available online: https://www.aatbio.com/tools/ic50-calculator; accessed on 21^st^ June 2021) on the basis of the cell viability ratio between treated and control cells. The subsequent biological assays were performed with CF extracts at the IC_50_ achieved at 24 (IC_50_24) and 48 h (IC_50_48), unless otherwise indicated. 

### Wound healing assay

The scratch/wound healing assay is commonly used to examine the modulation of cell locomotory ability *in vitro* (Luparello et al., 2020[41]). This experiment was performed as described by Nelson et al. (2014[54]). Briefly, HepG2 cells were seeded in 6-well plates and, once sub-confluent, the monolayer was scraped three times in parallel with a 200 µL pipette tip and a perpendicular line was drawn with a permanent marker. The culture medium was replaced with either plain medium (control) or medium containing CF extracts at IC_50_24 or at a lesser concentration, i.e. 5 µg/mL, as reported for the evaluation of the wound healing potential of the CF from *Astropecten indicus* sea star on human lung carcinoma cells (Baveja et al., 2018[4]). Selected sites of intersection between the scratched monolayer and the drawn line were photographed under a phase-contrast microscope at time intervals up to 24 h from the start of the assay. Wound healing area measurements were performed on the acquired images using the ImageJ/Fiji® plug-in developed by Suarez-Arnedo et al. (2020[63]).

### Flow cytometry

For each analysis, three independent flow cytometric assays were performed on treated and control cells as previously described (Luparello et al., 2019[43][39], 2020[42]), using a FACSCanto instrument (BD Biosciences, Franklin Lakes, NJ, USA). Ten thousand events were assessed and the obtained data were analyzed with the Floreada analysis tool available at https://floreada.io (accessed in May 2021). Gating in the FSC *vs.* SSC plot was performed to exclude debris, which displayed low FSC values, whereas gating in the FSC-H *vs.* FSC-A plot was performed to exclude doublets and multiplets in cell cycle analyses.

#### Cell cycle analysis

For the evaluation of cycle phase distribution, cells were fixed with cold 70 % ethanol, treated with 40 µg RNase A/mL, and stained with 20 µg propidium iodide/mL.

#### Apoptosis analysis

As a hallmark of apoptosis onset, the externalization of phosphatidylserine was evaluated with the Annexin V-FITC kit (Miltenyi Biotec, Bergisch Gladbach, Germany) according to the manufacturer's instructions.

#### Transmembrane mitochondrial potential (MMP) analysis

The MMP was checked using the JC1 dye (Molecular Probes, Eugene, OR, USA) (Librizzi et al., 2012[31]), which exhibits potential-dependent accumulation in intact mitochondria where the compound undergoes a fluorescence emission shift from green (~529 nm) to red (~590 nm). Thus, in the case of dissipation of MMP, a decrease in the red/green fluorescence intensity ratio can be observed. As a positive control, cells were treated with 1 µM valinomycin, a mitochondria-depolarizing K^+^ ionophore.

#### Reactive oxygen species (ROS) production analysis

The production of ROS was evaluated using the ROS Detection Assay Kit (Canvax Biotech, Cordoba, Spain) which contains the cell-permeant reagent dichlorodihydrofluorescein diacetate (H_2_DCFDA), an indicator of reactive oxygen intermediates such as COO- and ONOO-, that becomes fluorescent when oxidized, following the manufacturer's instructions. 

#### Acidic vesicular organelle (AVO) accumulation analysis

Double membrane-AVOs are typically developed by cells undergoing autophagy (Murugan and Amaravadi, 2016[51]; Luparello, 2021[38]). Changes in intracellular AVO accumulation were checked by staining with acridine orange, a green fluorescent dye that, once taken up by acidic vesicles and protonated, forms aggregates that show red fluorescence. For this purpose, flow cytometric analysis was performed on the control and treated cells stained with 100 µg acridine orange/mL (Sigma) for 20 min in the dark after fixation with cold 70 % ethanol. 

### Statistical analysis

Data were analyzed with analysis of variance (ANOVA) using SigmaPlot 11.0 software (SYSTAT, San Jose, CA, USA). A *p*-value < 0.05 was considered statistically significant. 

## Results

### CFE inhibits HepG2 cell viability and affects cell cycle progress 

**Figure 2:**
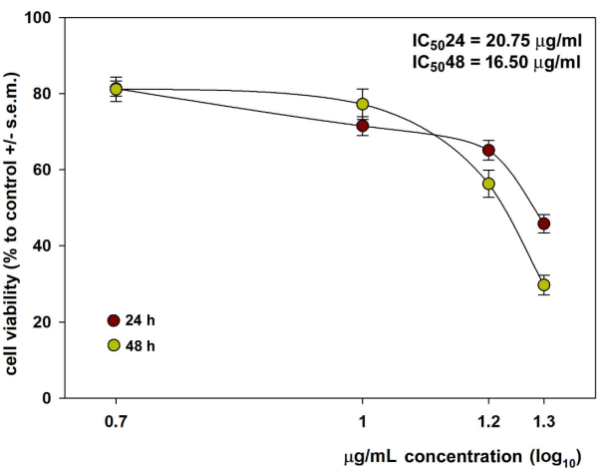
Dose-response effect of CFE from *H. tubulosa* at 2.5, 5, 10 and 15 µg/ml concentration on the viability of HepG2 cells after either 24 (brown circles) or 48 h (green circles) of exposure. Error bars correspond to the standard error of the mean (s.e.m.) of three independent measurements. *P *values comparing viability ratios to controls were < 0.05 for every measurement.

In the first set of experiments, we checked the effect of dose- and time-dependent incubation with the aqueous CFE from *H. tubulosa *on HepG2 cell viability *via* an MTT assay. As shown in Figure 2(Fig. 2), the number of CFE-treated viable cells decreased in a protein concentration-dependent manner both after 24 and 48 h exposures with an average IC_50_24 and IC_50_48 of 20.75 and 16.5 µg/mL, respectively. Once the cytotoxic potential of the preparation was confirmed, all subsequent assays aimed to reveal more detailed data on the biological mechanism of CFE-induced perturbations were carried out by exposing HepG2 cells at the IC_50_24 and IC_50_48 of the CFE.

**Figure 3:**
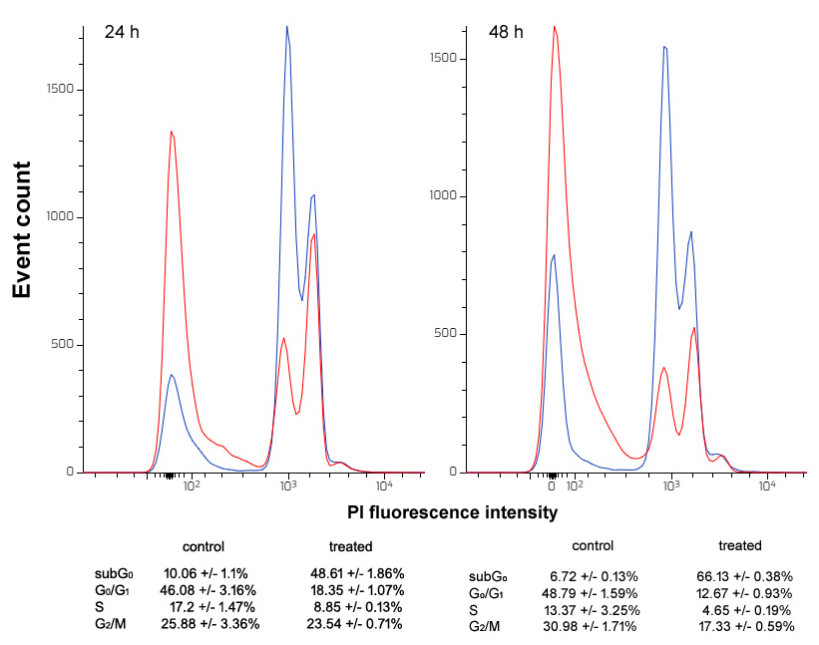
DNA profiles of control (blue line) and CFE-treated (red line) HepG2 cells after 24 and 48 h of exposure to the IC_50_24 and IC_50_48 of the preparation, respectively. Cell cycle distribution is reported in the annexed tables for both cell samples (mean ± s.e.m. of three independent experiments).

Then, CFE-exposed HepG2 cells were tested for the distribution of the cell population in the cell cycle phases. As shown in Figure 3(Fig. 3), 24 h of exposure to the IC_50_24 decreased the percentage of G_0_/G_1_ and S phase cells (control *vs.* treated = 46.08 % *vs.* 18.35 % and 17.2 *vs*. 8.85 %, respectively) and increased the sub-G_0_ population (control *vs.* treated = 10.06 % *vs.* 48.61 %) suggesting a restrained progression into a new cell cycle after mitosis required to activate repair mechanisms or apoptosis and an accumulation of cells with less bright or fragmented DNA, i.e. necrotic or apoptotic. The additional impairment of the G_2_/M phase fraction observed after 48 h of treatment with the IC_50_48 of the CFE (control *vs.* treated = 30.98 % *vs.* 17.33 %), the further decrease of the percentage of G_0_/G_1_ and S phase cells (control *vs.* treated = 48.79 % *vs.* 12.67 % and 13.37 % *vs*. 4.65 %, respectively) and the increase in the percentage of cells in the sub-G_0_ fraction (control *vs.* treated = 6.72 % *vs.* 66.13 %) was indicative of prominent cell cycle arrest and cell death as a result of prolonged exposure.

### CFE increases the percentage of apoptotic HepG2cells

**Figure 4:**
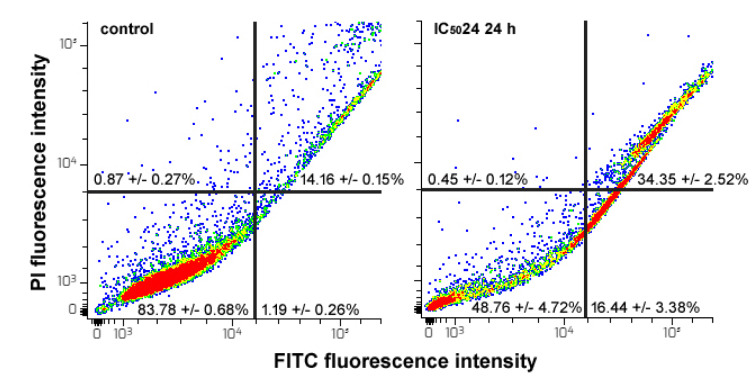
Flow cytometric assays for apoptosis in HepG2 cells cultured in control conditions or exposed to CFE IC_50_24 for 24 h. The plots show the results of representative experiments and the percentages, indicated as the mean ± s.e.m. of three independent experiments, refer to viable annexin-V^-^/PI^-^ cells (bottom left quadrant), early apoptotic annexin-V^+^/PI^-^ cells (bottom right quadrant), late apoptotic annexin-V^+^/PI^+^ cells (top right quadrant) and necrotic annexin-V^-^/PI^+^ cells (top left quadrant).

To determine whether the observed impairment of cell cycle progress could be at least in part ascribed to an apoptosis-promoting effect of the CFE on HepG2 cells, exposed and control samples were assayed for the externalization of phosphatidylserine using recombinant annexin-V conjugated to green fluorescent FITC dye in conjunction with propidium iodide (PI) as an indicator of cell viability. As shown in Figure 4(Fig. 4), the flow cytometric data indicated that after 24 h of exposure to the IC_50_24 of the CFE the proportion of viable cells decreased from about 83 % of the controls to about 48 %. In contrast, the percentage of late apoptotic cells (annexin-V^+^/PI^+^) increased from about 14 % of the controls to about 34 %, whereas that of early apoptotic cells (annexin-V^+^/PI^-^) increased from about 1 % of the control to about 16 %. This result is in line with the previous data regarding the amount of the sub-G_0_ cell population after 24 h of exposure to the CFE. No significant difference was found between the control and annexin-V^-^/PI^+^ cells, commonly regarded as the necrotic population. 

### CFE provokes the dissipation of MMP and the initial up-regulation of ROS in HepG2 cells 

**Figure 5:**
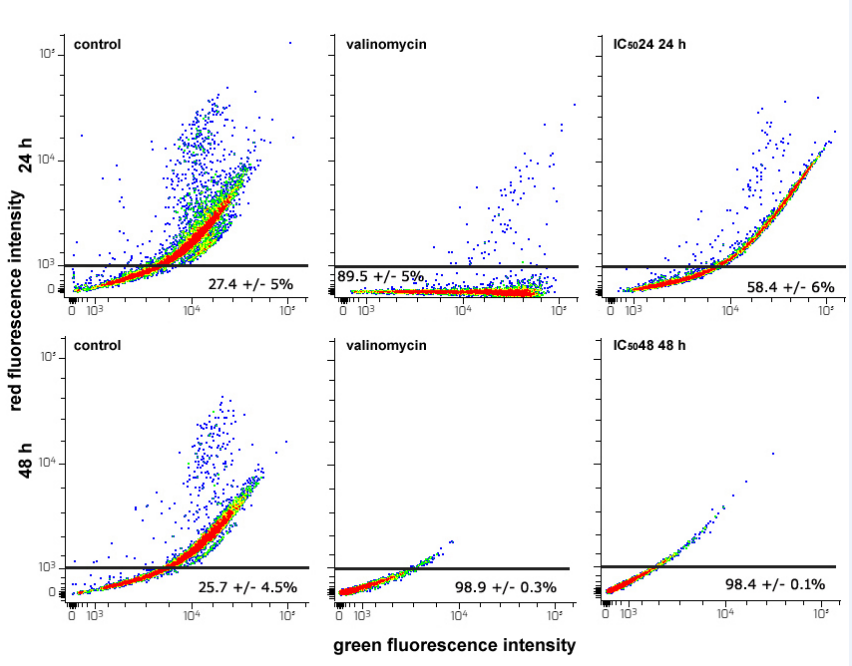
Flow cytometric assays for MMP in HepG2 cells cultured for 24 or 48 h in control conditions, in the presence of 1 µM valinomycin, and of either CFE IC_50_24 or CFE IC_50_48. The plots show the results of representative experiments and the percentages in the bottom quadrants of each frame, indicated as the mean ± s.e.m. of three independent experiments, are referred to dim red-emitting cells that underwent MMP dissipation.

Extensive damage to the mitochondria may lead cells to apoptotic death (Ly et al., 2003[44]). However, a strong positive correlation was observed between the MMP and the production of ROS which are known to act as signaling molecules in a plethora of intracellular pathways regulating both cell survival/proliferation and cell death (Zhang et al., 2016[75]; Gao et al., 2020[18]). To detect variation of MMP in CFE-exposed HepG2 cells we used the mitochondria-selective JC1 probe and evaluated the quantity of cells with bright green/bright red emission (endowed with intact MMP) and those with bright green/dim red emission (affected by MMP collapse) in control and treated preparations. CFE caused the dissipation of MMP. In particular, as shown in Figure 5(Fig. 5), the percentage of dim red-emitting cells increased from about 27 and 25 % of the controls to about 58 and 98 % in cells incubated with IC_50_24 CFE for 24 h and IC_50_48 CFE for 48 h, respectively. In the latter case, this proportion was very similar to that of the valinomycin-exposed positive control. 

**Figure 6:**
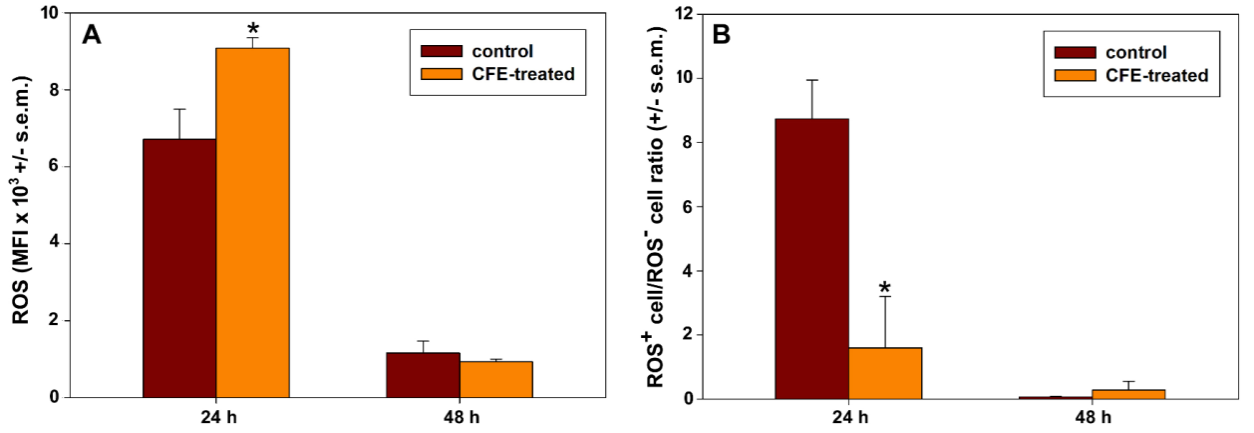
Bar graphs showing the ROS-associated MFI in ROS^+^ cell subpopulations (A) and the ROS^+^ cell/ROS^-^ cell ratio (B) in parallel preparations of control and IC_50_24 and IC_50_48 CFE-treated HepG2 cells for 24 and 48 h (mean ± s.e.m. of three independent experiments). * *p* < 0.001 (A) and = 0.029 (B) determined with independent t-tests.

The ability of the CFE to affect mitochondrial metabolism was also determined by evaluating the accumulation of ROS. To detect variation in ROS production in CFE-exposed HepG2 cells we used the H_2_DCFDA probe and evaluated its oxidation to green-emitting DCF by various peroxide-like and nitric oxide-derived reactive molecules. In each sample, the flow cytometric data revealed the presence of two distinct cell subpopulations endowed with low (ROS^-^) and high rates (ROS^+^) of ROS generation. The mean fluorescence intensity (MFI) values of the events associated with the ROS^+^ subpopulations were recorded to compare their rates in the different experimental conditions. In addition, the percentage of ROS^+^ cells within the whole populations was calculated. Taking the data reported in Figure 6A and B(Fig. 6) into account, a transient up-regulation in ROS generation could be observed after 24 h incubation with the CFE with a 1.45-fold ROS increase in exposed *vs*. control cells (control cells' MFI = 6727, treated cells' MFI = 9770; *P*< 0.05). On the other hand, the ROS^+^ cell amount was smaller than that of controls (about 18 %) after 24 h of treatment with IC_50_24 CFE. This may suggest the initial occurrence of an increased intracellular ROS production coupled with an extended cell damage, both aspects that fit well with the already-reported significant decrease of MMP with consequent deterioration of cells' respiratory activity. As expected from the progressive advancement of cellular impairment and death, HepG2 cells exposed to IC_50_48 CFE for 48 h showed a drastic arrest of ROS overproduction *vs.* control cells (control cells' MFI = 1166; treated cells' MFI = 960; no statistical significance).

### CFE impairs HepG2 cell autophagic behavior

**Figure 7:**
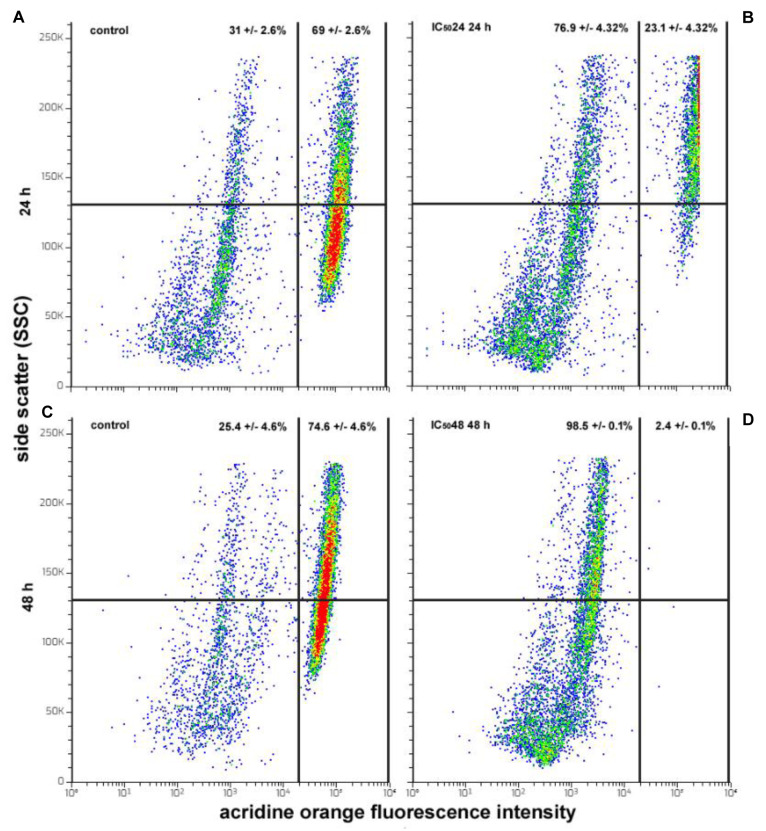
Flow cytometric assays for AVOs in HepG2 cells cultured in control conditions for 24 (A) and 48 h (C) or exposed to CFE IC_50_24 for 24 h (B) and CFE IC_50_48 for 48 h (D). The plots show the results of representative experiments and the percentages, indicated as the mean ± s.e.m. of three independent experiments, refer to AVO^+^ cells (right quadrants) and AVO^-^ cells (left quadrants).

To determine whether the CFE could modify the autophagic behavior of HepG2 cells, acridine orange staining was performed to label AVOs, a hallmark of autophagic cells. In fact, as reported by Gibson (2013[19]), flow cytometric scanning of changes in the amount of AVOs is indicative of variations in autophagosome accumulation and autolysosome formation. Analysis of the plots revealed the constant presence of two distinct cell subpopulations characterized by low (AVO^-^) and high rate (AVO^+^) of acridine orange fluorescence. Consistent with the data from Sun et al. (2018[66]), these results confirmed that HepG2 cells are endowed with a high basal level of autophagy, with the percentage of AVO^+ ^actively-autophagic cells being about 69 and 74 % at 24 and 48 h of culture, respectively, under control conditions (Figure 7A, C(Fig. 7)). However, the proportion of AVO^+^ cells decreased 24 h after treatment with IC_50_24 CFE (25 %; Figure 7B(Fig. 7)). Further incubation for 48 h with the IC_50_48 indicated the almost complete disappearance of the AVO^+ ^cell population, whose proportion dropped to about 2 % (Figure 7D(Fig. 7)).

### CFE inhibits HepG2 cell migration in vitro

**Figure 8:**
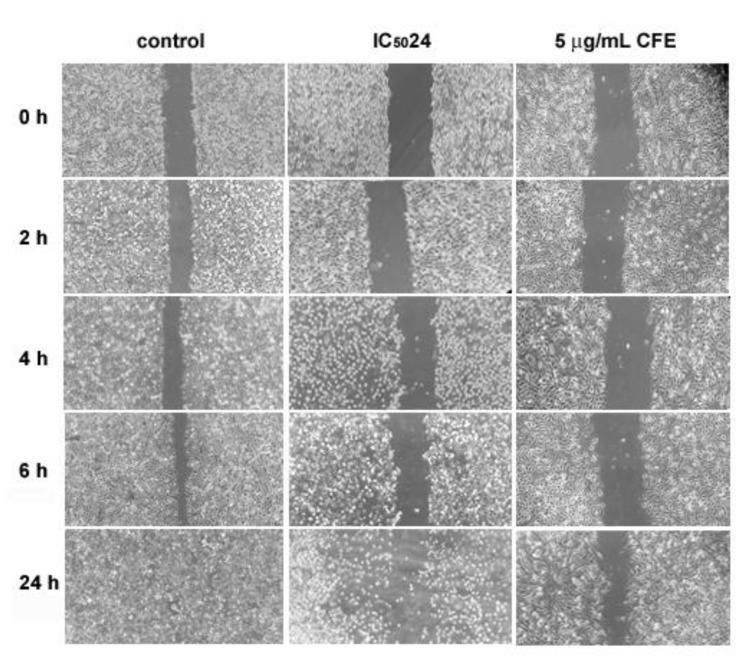
Representative phase-contrast micrographs acquired during wound-healing experiments at different time intervals under control conditions, and in the presence of CFE at IC_50_24 and 5 µg/mL concentration. The assay was performed in triplicate. Microscopic magnification = 20X

Wound healing assays were performed and monitored within 24 h to examine the effect of incubation with the aqueous CFE from *H. tubulosa *on HepG2 cell locomotory ability. As illustrated by Figure 8(Fig. 8), under control conditions, HepG2 cell migration determined the advancing reduction of the denuded area (mean area % = 23 at time 0), which had already started at 2 h from the scratch time (mean area % = 17) leading to partial obliteration after 6 h (mean area % = 6) and then total closure of the wound within 24 h. In contrast, exposure to IC_50_24 CFE inhibited cells' ability to migrate into the scratch area, and, as expected, cells in the last period of treatment displayed signs of suffering and damage such as rounding and detaching from the substrate. Based on the data by Baveja et al. (2018[4]) who demonstrated a non-cytotoxic and effective wound healing effect of 24 h-administration of 5 µg/mL of the CFE from another invertebrate species, the sea star *A. indicus*, to human A-549 tumor cells, we also tested HepG2 cells' migratory behavior in the presence of *H. tubulosa*'s CFE at low concentration. As revealed in the panel, although the cells' healthy morphological appearance appeared unmodified by the treatment, a slight sign of reduction in the scratch area was observed only at 24 h after exposure (mean area % at time 0 and after 24 h = 24 and 19, respectively), thereby confirming the migration-inhibiting effect exerted by *H. tubulosa*'s CFE on liver cancer cells. 

### Proteomic profile of the CFE identifies potential contributors to the observed cytotoxic activity 

It is widely acknowledged that peptides and proteins produced by marine invertebrates may be endowed with cell death-promoting properties, such as the ability to induce apoptosis, activate the mitochondrial intrinsic pathway and/or impair signal transduction pathways as well as cytoskeletal dynamics (Zheng et al., 2011[76]). Based on the observed cytotoxic role played by the CFE from *H. tubulosa* on HepG2 tumor cells, proteomic analysis of the aqueous preparations was performed after proteolysis of the samples and MS sequencing to detect bioactive components. Overall, 1648 obtained spectra matched forward peptides and the final output reported 115 forward and 20 reverse proteins and 321 unique forward peptides. The estimated spectrum-level FDR on true proteins was 0.1 %. A bioinformatic similarity search against the different databases identified 18 proteins contained in the CFE that are potentially associated with the various aspects related to the impairment of HepG2 cell biological activities reported in the previous paragraphs, as well as with exosome secretion. The peptide sequences and the results of alignments selected on the basis of sorting by the best E value are reported in Table 1(Tab. 1).

## Discussion

Holothurians are known to be exploited for human food purposes and as a remedy in traditional medicine, and in the present paper we determined whether the water-soluble CFE from *H. tubulosa* could exert any cytotoxic effect against an *in vitro* cell model of a cancer of the digestive system, i.e. hepatocellular carcinoma. Our data depicted an anti-tumoral role of the preparation, at least under the conditions used, characterized by an early block of the cell cycle at the G_2_/M phase coupled to oxidative stress promotion, AVO depletion and mitochondrial dysfunction and leading to apoptotic death. 

G_2_/M arrest is an event commonly preceding apoptosis (Chen et al., 2021[9]; Tawfik et al., 2021[69]) which, as reviewed by DiPaola (2002[14]), may be based upon G_2_ checkpoint impairment or problems in the assembly and dynamics of mitotic spindle structure, an aspect that requires further investigation. Elevated levels of basal autophagy in cancer cells, such as HepG2, are instrumental for enabling their survival and active growth and locomotion by fulfilling the high metabolic and energetic demands (Luparello, 2021[38]). Thus, a possible mechanism of CFE cytotoxicity may involve the suppression of “protective” autophagy which, as reported by Zhu et al. (2021[77]) for chitooligosaccharide-treated HepG2 cells, leads to the inhibition of cell proliferation and apoptosis *via* the intrinsic pathway as suggested in our model system by the collapse of MMT. Mitochondria are both primary sources and targets of ROS (Marchi et al., 2012[45]). However, HepG2 cells express low levels of cytochrome P450 family 2 subfamily E member 1 (CYP2E1), a ROS-generating enzyme of the endoplasmic reticulum, and therefore are a useful model to check the formation of ROS mainly from mitochondrial sources (Jiang et al., 2015[24]). Thus, CFE-triggered respiratory deficiency might affect mitochondrial ATP synthesis, and the impairment of the respiratory chain and the oxidative phosphorylation system could stimulate the leakage of electrons from the transport chain with consequent up-regulation of ROS production. In turn, this could establish a vicious cycle of an oxidative stress-induced increase of mitochondrial damage, also at the transcriptome level, with consequent further ROS production. Additionally, impairment of the autophagic flux, which is a common mechanism for the clearance of damaged ROS-overproducing mitochondria, may participate in this vicious cycle and contribute to cell death triggering (Kongara and Karantza, 2012[27]). The derangement of HepG2 cells' healthy state appeared also responsible for an early block of cell locomotory behavior, even at a CFE dose less than the IC_50_24, thereby suggesting that the preparation may be a potential suppressor of HCC metastatic attitude, even at low concentrations.

The biochemical compositions of extracts from the isolated tegument and dried body of *H. tubulosa *have been the object of some publications that have also provided evidence of the anticancer and antioxidant potential of the preparations (Alper and Güneş, 2020[1]; Zmemlia et al., 2020[81]). To the best of our knowledge, no study is available in the literature on the biochemical composition of the coelomic fluid of *H. tubulosa*, which represents a significant portion of its mass and contains a complex mixture of soluble molecules secreted constitutively by different parts of the invertebrate's body. Therefore, in search of molecular constituents that may conceivably be involved in the observed cytotoxic activity, we complemented cellular assays with a first-released proteomic analysis of the CFE. This study predicted a number of putative anticancer proteins responsible for the lethal effect against HepG2 cells. In parallel, exosome-linked signatures were also detected suggesting the release of extracellular vesicles carrying, among the others, the intracellular constituents likely causing the anti-HepG2 effects in the coelomic fluid of *H. tubulosa*. Exosomes are conceivably preserved intact by the method of preparation of the CFE and therefore could be ready to fuse with and transfer their cargo into cancer cells. Notably, the presence of exosomes in the coelomic fluid of aquatic invertebrates has been acknowledged and related to their involvement in cell-to-cell communication and immune defence (Chiaramonte et al., 2014[11]; Auguste et al., 2020[2]; D'Alessio et al., 2021[13]).

Among the protein signatures identified, some may be mainly, but not only, associated with the activation of programmed cell death. Deleted In Malignant Brain Tumors 1 (DMBT1) is a tumor growth suppressor and an effector of genetic resistance to hepatocarcinogenesis in rats and humans (Frau et al., 2012[17]). Data obtained with the GBC-SD gallbladder cancer cell line demonstrated that its overexpression can down-regulate cell proliferation and induce apoptosis *via *stabilization of the phosphatase and tensin homolog (PTEN) and inhibition of the PI3K-Akt pathway, reducing xenograft tumor growth *in vivo* (Sheng et al., 2019[61]). Similarly, expression of the huntingtin-interacting protein 1-related protein, a component of the clathrin**-**mediated endocytosis pathway, stimulated apoptosis and inhibited proliferation, locomotion and invasion of gastric tumor cells by acting on the Akt pathway (Zhu et al., 2020[79]). The invertebrate semaphorin-1A is known to be similar to class-6 semaphorins of vertebrates (Battistini and Tamagnone, 2016[3]), whose tumor-suppressing and apoptosis-stimulating roles have been recognized in different cancer cell models (Lu et al., 2012[37]; Fard and Tamagnone, 2021[16]). Peptides from the beta-1,3-glucan binding protein, a pattern recognition protein against invasive pathogens, of the pacific abalone *Haliotis discus hannai* showed pro-apoptotic properties against human cervical, lung and colon cancer cells (Nam et al., 2016[52]). Up-regulation of the apoptosis-stimulating of p53 protein 1 in HepG2 cells exposed to the all-trans retinoic acid derivative 4-amino-2-trifluoromethyl-phenyl retinate was proven to result in cell cycle arrest and apoptosis (Liu et al., 2016[32]). The zinc finger C3H1 domain-containing protein, which is known to modulate N6-methyladenosine RNA modification, has been considered a tumor suppressor correlated with a better survival rate in hepatocellular carcinoma (Huang et al., 2020[22]). Conceivably, this might be based upon its interference with K-ras and ERK signaling as reported by Zhu et al. (2019[78]) who observed the inhibition of proliferation and invasion of zinc finger C3H1 domain-containing protein over-expressing colorectal tumor cells. In breast cancer cell models, the up-regulation of galactosylceramide sulfotransferase, which increases the release of sulfatides from the sphingolipid, was found to be associated with the onset of programed cell death due to the decrease in the amount of the latter acting as an anti-apoptotic molecule (Suchanski et al., 2018[64]). In the same models, the over-expression of ependymin-related protein 1, a poorly characterized transmembrane protein, was also shown to inhibit cell proliferation and migration leading to apoptosis *via *activation of the p53 signaling pathway (Liang et al., 2020[30]). Ultimately, over-expression of *PRPF39*, coding for pre-mRNA-processing factor 39, in HepG2 cells appeared related to the acquisition of enhanced sensitivity to the cytotoxic action of cisplatin (Qin et al., 2021[58]). This was ascribed, at least in part, to its acknowledged negative regulation on the expression of *TFDP2* coding for E2F dimerization partner 2 involved in the progression of the cell cycle, and positive regulation on the expression of *MAP3K4* coding for mitogen-activated protein kinase kinase kinase 4 which participates in apoptosis-promoting intracellular signaling (Stark et al., 2012[62]). 

In addition, other protein signatures may be mainly associated with the inhibition of cell migration, thus being related to the anti-metastatic property of the CFE preparation. In particular, the H3 chain of the inter-alpha trypsin inhibitor, an extracellular matrix component that binds to vitronectin, fibronectin and hyaluronic acid thereby influencing their biological activity (Lord et al., 2020[36]), induces a significant decrease in the number of lung metastases in H460M cell-injected mice probably due to the increase in the cell adhesion rate (Paris et al., 2002[56]). In addition, Sur-Erdem et al. (2020[67]) reported that the giant actin-binding protein nesprin-1 is able to reverse the tumorigenic phenotype of HuH7 hepatocellular carcinoma cells, whereas data from Huang et al. (2016[21]) demonstrated that up-regulation of endoplasmic reticulum resident protein 44 determines the inhibition of human A549 lung cancer cell migration *via *an inositol 1,4,5-triphosphate receptor‐dependent pathway. Notably, over-expression of the serum protein ficolin-2 was proven to significantly reduce the migratory and invasive behavior of liver cancer cells *in vitro *and *in vivo*, attenuating the epithelial-mesenchymal transition *via *stimulation of the TGFβ/Smad transduction pathway (Yang et al., 2016[74]).

Finally, one of the identified protein signatures found may relate to the modulation of the autophagic flux. In fact, in retinal cells TBC1 domain family member 17, a RAB GTPase-activating protein, has been shown to impair autophagy, as well as the trafficking and recycling of transferrin receptors, both leading to the loss of cellular homeostasis and promotion of cell death (Chalasani et al., 2014[8]).

Among the proteins listed, ficolin-2, semaphorin and putative endoplasmic reticulum resident protein 44 were reported to be contained in human and murine exosomes and released into body fluid (Looze et al., 2009[35]; Biswas et al., 2019[5]; Xia et al., 2021[73]). Furthermore, our proteomic analysis of *H. tubulosa*'s CFE revealed the presence of histones H1 and H2B which can be considered additional markers of the nanovesicles (Auguste et al., 2020[2]). In fact, extracellular histones are involved in exosome-mediated adhesion and could conceivably be involved in exosome uptake by target cells and the subsequent activation of signaling (Nangami et al., 2014[53]; Muhsin-Shrafaldine et al., 2016[50]; Ochieng et al., 2018[55]).

In summary, we have shown that *H. tubulosa*'s CFE is cytotoxic towards hepatocellular carcinoma cells *in vitro*. A limitation of the present study is the lack of isolation and identification of the CFE component(s) to which the observed effects may be attributed. However, the proteomic profile analysis revealed a number of proteins that can seemingly play anti-cancer roles at different levels. Based on data from the literature (e.g. Khotimchenko, 2018[26]) the contribution of varied sea cucumber-derived molecules, falling into the categories of triterpenes, glycosaminoglycans and cerebrosides, among others, cannot be excluded, as well as the existence of potential and complex synergic activities between these compounds. The data reported here strongly suggest that the water-soluble anti-HepG2 cell constituent(s) of the CFE is/are resistant to lyophilization, resuspension, and freeze-thawing cycles.

## Conclusions

It is widely acknowledged that additives may be added to food directly or incorporated into the aliments in trace amounts during packaging, storage, or handling (Karunarathne et al., 2020[25]). As reported by Vilela et al. (2018[72]), packaging technologies are being developed to improve the safety and maintain the nutritional quality of minimally-processed food *via *the anti-microbial, anti-oxidant, and anticancer properties of the additives. Moreover, products from marine sources have great potential to be used as barrier coatings for multilayer packaging or edible coatings directly applied to food. In conclusion, considering these emerging technologies, the “active constituent(s)” present in the samples under study merit further investigation aimed at developing novel promising prevention and/or treatment agents and beneficial supplements for the formulation of functional food and food-packaging material.

## Declaration

### Author contributions

Conceptualization, CL and MV; methodology, RB, LD, MM and VD; investigation, RB, GA, VL, LD and MM; data curation, CL; writing-original draft preparation, CL; writing-review and editing, CL and MV; supervision, CL, VA and MV; funding acquisition, VA. All authors have read and agreed to the published version of the manuscript.

### Ethics approval 

The "Assessorato regionale dell'agricoltura, sviluppo rurale e pesca mediterranea” of Sicily has approved the use of *Holothuria* in this study (authorization no. 87468 of 25.10.2021).

### Conflict of interest

The authors declare no conflict of interest.

### Acknowledgments 

This work was supported by the University of Palermo (Italy) [grant Fondo Finalizzato alla Ricerca (FFR) 2021] to C.L. and M.V.

## Figures and Tables

**Table 1:**
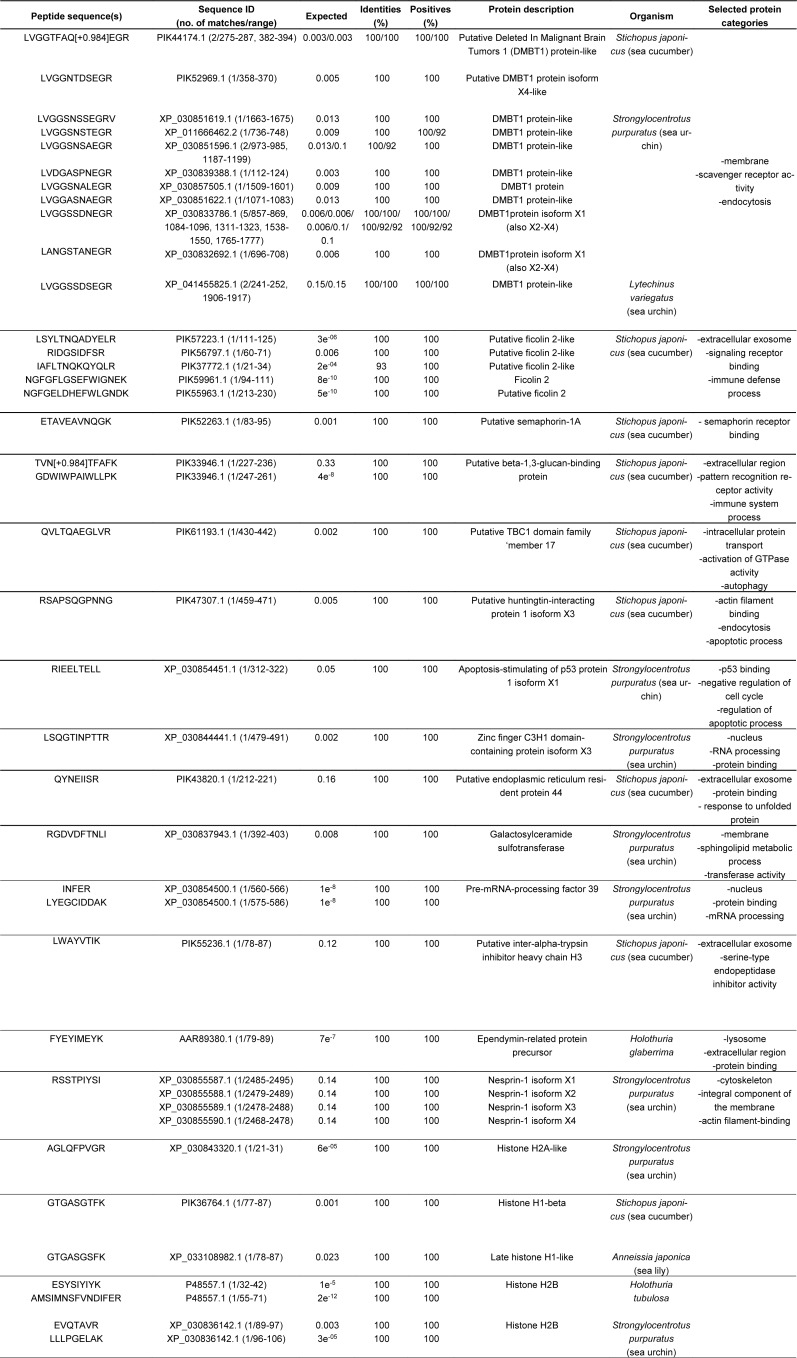
Cytotoxic activity-associated protein profile of the CFE from *H. tubulosa*
